# GLP-1 Increases Circulating Leptin Levels in Truncal Vagotomized Rats

**DOI:** 10.3390/biomedicines11051322

**Published:** 2023-04-28

**Authors:** Tiago Morais, Sofia S. Pereira, Sara Andrade, Diogo Neves, Marta Guimarães, Mário Nora, Marcos C. Carreira, Felipe F. Casanueva, Mariana P. Monteiro

**Affiliations:** 1 Endocrine and Metabolic Research, UMIB-Unit for Multidisciplinary Research in Biomedicine, ICBAS-Instituto de Ciências Biomédicas Abel Salazar, Universidade do Porto, 4050-313 Porto, Portugal; 2 Laboratory for Integrative and Translational Research in Population Health (ITR), University of Porto, 4050-313 Porto, Portugal; 3 Department of General Surgery, Centro Hospitalar de Entre o Douro e Vouga, 4520-220 Santa Maria da Feira, Portugal; 4 CIBER de Fisiopatologia Obesidad y Nutricion (CB06/03), Instituto Salud Carlos III, 15706 Santiago de Compostela, Spain; 5 Department of Medicine, USC University Hospital Complex, University of Santiago de Compostela, 15705 Santiago de Compostela, Spain

**Keywords:** leptin, GLP-1, vagotomy, rat, adipose tissue

## Abstract

GLP-1 is a gastro-intestinal hormone acting within the gut/brain axis for energy balance regulation. We aimed to evaluate the role of the vagus nerve in whole-body energy homeostasis and in mediating GLP-1 effects. For this, rats submitted to truncal vagotomy and sham-operated controls underwent a comprehensive evaluation, including eating behavior, body weight, percentage of white (WAT) and brown adipose tissue (BAT), resting energy expenditure (REE) and acute response to GLP-1. Truncal vagotomized rats had significantly lower food intake, body weight, body weight gain, WAT and BAT, with a higher BAT/WAT ratio, but no significant difference in REE when compared to controls. Vagotomized rats also had significantly higher fasting ghrelin and lower glucose and insulin levels. After GLP-1 administration, vagotomized rats depicted a blunted anorexigenic response and higher plasma leptin levels, as compared to controls. However, in vitro stimulation of VAT explants with GLP-1 resulted in no significant changes in leptin secretion. In conclusion, the vagus nerve influences whole-body energy homeostasis by modifying food intake, body weight and body composition and by mediating the GLP-1 anorectic response. The higher leptin levels in response to acute GLP-1 administration observed after truncal vagotomy suggest the existence of a putative GLP-1-leptin axis that relies on the integrity of gut–brain vagal pathway.

## 1. Introduction

The process of energy balance depends on the integrity of the gut/brain axis, where the vagus nerve acts as the major neuroanatomical link between the gastro-intestinal (GI) tract and the brain [1]. Therefore, the vagus nerve is also recognized to play a major role in appetite regulation [2,3]. Highlighting its importance in weight loss processes, vagotomy was demonstrated to limit weight loss after gastric bypass in rodents [4,5] and humans [6], suggesting that post-surgical weight loss may also depend on the vagal pathway integrity.

Although its precise role in energy homeostasis remains to be fully established, the vagus nerve was shown to express several GI hormone receptors, including the receptors for glucagon-like peptide-1 (GLP-1) [7], ghrelin [8] and leptin [9]. In fact, the inhibitory effect of several GI hormones on food intake was demonstrated to be abolished after vagus nerve sectioning [2].

GI hormones are at the forefront of the novel weigh loss interventions [10,11]. In fact, a GLP-1 analogue is already licensed for obesity treatment [12]. GLP-1 is a product of the proglucagon gene, which is secreted by intestinal L cells into the hepatic portal circulation in response to nutrient ingestion [13].

GLP-1 is best known for its incretin effect, whereby it augments glucose-stimulated insulin secretion by pancreatic β cells [14], which has prompted the successful development of GLP-1 based therapies for type 2 diabetes treatment [15]. Additionally, GLP-1 is produced in the caudal nucleus of the solitary tract and in the ventrolateral medulla [16], while GLP-1 receptors (GLP1R), in addition to being expressed in the vagus nerve, are also expressed in the hindbrain and in hypothalamic nuclei, which are anatomical sites that participate in the regulation of energy homeostasis [17]. Thus, the anatomy of the GLP-1 system suggests that this molecule has diverse roles in regulating the body’s energy balance.

In rodents and humans, GLP-1 and its analogs inhibit food intake after acute administration [2,18] and reduce body weight when delivered chronically [19]. These effects seem to be partially mediated via the vagus nerve, given that vagotomy was previously reported to blunt the anorectic effects of GLP-1 [2], despite other GLP-1 effects that could be mediated by vagal independent mechanisms as well [20].

The primary aim of the present study was to assess the role of the vagus nerve in mediating the influence of acute GLP-1 administration on whole-body energy homeostasis.

## 2. Materials and Methods

### 2.1. Vagotomy Procedure

All animal studies were done in compliance with local and European legislation (86/609/EC) and duly approved by the local ethics board (Direção-Geral da Alimentação e Veterinária; 0420/000/000/2011, 8 November 2011).

Wistar Rats (male, weighing between 300 to 400 g) were placed in individual cages with free access to food and water through the experimental period. Cages were kept under controlled temperature conditions (21–23 °C) and with 12 h light cycles.

Rats were allowed one week of acclimatization to housing conditions. Two weight-matched groups (*n* = 12 per group) were formed via a randomization procedure, with one group being subjected to bilateral sub-diaphragmatic truncal vagotomy and the other to sham surgery.

Prior to surgical procedure, rats were fasted for 12 h, weighed and anaesthetized with sevoflurane (Abbott Laboratories Limited, Maidenhead, UK) (8% for induction, 3% for maintenance). Surgical procedure was performed as previously described [2,21].

Post-operatively, antibiotic prophylaxis was administered, via intraperitoneal (ip) injection, consisting of amoxycillin combined with flucoxicillin (2.5 mg/mL) (Labesfal, Lisboa, Portugal). Rats’ body weight and food intake were monitored daily throughout the remainder of the study. Animals were allowed to recover from surgery before further experimental procedures.

### 2.2. Confirmation of the Vagotomy Procedure

The importance of acclimatization to the experimental procedures in the feeding response to GI hormones has been previously documented [22]. Therefore, rats were handled on a daily basis from 5 days post-surgery and familiarized to the experimental procedures. To confirm that the vagus nerve ablation was successful, a procedure based on the feeding response to cholecystokinin octapeptide (CCK-8) (10 µg/kg ip) (Bachem, St Helens, UK) was used as previously described [2,21]. Further confirmation of vagotomy success or failure was obtained by weighing the rats’ stomachs after the sacrifice. As previously described [23], a 2-fold increase in wet stomach size can be considered a marker of a successful vagotomy.

Rats in the vagotomized group that exhibited an anorectic response to CCK-8 (inhibition of food intake of 50% or more) when compared to the saline control and with a stomach weight less than 2-fold greater as compared to the average stomach weight of sham controls were excluded from analysis (16.6% vagotomized rats were excluded).

### 2.3. Resting Energy Expenditure Assessment and Feeding Studies

Resting energy expenditure (REE) was assessed by indirect calorimetry, as previously described [21].

Rats were fasted overnight prior to GLP-1 (7–36) (165 µg/kg ip, *n* = 10) injection or saline (*n* = 12), in a crossover design, and food intake was measured as described above. The GLP-1 dosage administered was chosen according to a previously published dose-response study in which the effect was reported to be submaximal but still enough to significantly reduce food intake [2].

### 2.4. Experimental Endpoint

Rats from both experimental groups, vagotomized and sham-operated, were again randomly divided into 2 groups to be administrated with either GLP-1 (165 µg/kg) (Bachem, St Helens, UK) or saline 90 min prior to sacrifice with CO_2_ followed by exsanguination by cardiac puncture. EDTA tubes, kept at 4 °C, were used to collect blood, which was promptly centrifuged at 3000 rpm (10 min at 4 °C). Plasma was then collected and stored at −20 °C until further analyses. To assess total adiposity, epididymal white adipose tissue (EPI WAT) was collected and weighed.

### 2.5. Blood Glucose and Hormone Measurements

Blood glucose levels were determined using a commercially available glucometer (One Touch Ultra, Lifescan, Milpitas, CA, USA). Insulin resistance was calculated using the homeostatic model assessment for insulin resistance (HOMA-IR) formula:

HOMA-IR = (glucose [mg/dL] × insulin [mU/L])/405.

Concentrations of total and active GLP-1 (EZGLP1T-36K and EGLP-35K, Merck Millipore, Darmstadt, Germany), peptide YY (PYY) (S-1151, Bachem, Torrance, CA, USA), oxyntomodulin (OXM) (S-1392, Bachem, Torrance, CA, USA), insulin (EZRMI-13K, Merck Millipore, Darmstadt, Germany), total ghrelin (EZRGRT-91K, Merck Millipore, Darmstadt, Germany) and leptin (EZRL-83K, Merck Millipore, Darmstadt, Germany) were determined by ELISA using specific commercial kits according to the manufacturer instructions.

### 2.6. Hypothalamic Neuropeptide and UCP-1 Expression

At the time of sacrifice, basal hypothalamus and interscapular brown adipose tissue (BAT) were collected and immediately frozen by immersion in liquid nitrogen. Total RNA isolation and RNA expression analysis of NPY, AgRP, POMC and CART in the hypothalamus and of UCP-1 in BAT was performed as previously described [21].

### 2.7. In Vitro Stimulation of Human VAT Explants with GLP-1

Samples of visceral adipose tissue (VAT) were obtained from patients undergoing laparoscopic interventions in an elective setting. Details of the collection procedures have been described elsewhere [24]. A total of 19 subjects were enrolled and grouped based on body mass index (BMI) and glycemic status (Table S1). All participants provided informed consent prior to undergoing any study-related procedures. This study was approved by the Institutional Ethics Committee (CHEDV-HSS; CA 0830/16-Ot, 15 June 2016) and was conducted in accordance with the European ethical guidelines for medical research involving human subjects.

After the VAT was harvested, tissue fragments were promptly processed to remove any damaged tissue debris, weighed, cut up into smaller fragments of about 20 mg and were then placed in 48-well plates with 200 µL DMEM/F12 (12-719F, Lonza, Basel, Switzerland). Tissue was allowed to acclimate in a cell culture chamber (37 °C and 5% CO_2_) for two 1 h periods, with the tissue being washed with fresh media in between the two periods. Post-acclimatization period, VAT fragments were incubated with culture medium with culture media containing insulin (100 nmol/L, Actrapid, Novo Nordisk, Bagsværd, Denmark), 1% penicillin-streptomycin (P4333, Sigma-Aldrich, St. Louis, MO, USA) and 100 nmol/L of GLP-1 (Bachem, St Helens, UK). After 48 h, the VAT explant culture media was collected and stored at −20 °C for posterior analysis. Leptin levels were measured using the Human Leptin “Dual Range” kit (ELISA EZHL-80SK, Merck Millipore, Darmstadt, Germany).

### 2.8. Data and Statistical Analysis

Results are presented as mean ± standard error of the mean (mean ± SEM), except where explicitly indicated. Outliers were identified and removed using the ROUT method (Q = 5%). Normality was assessed using a Shapiro–Wilk test. Comparisons of two independent groups with normal distribution were carried out using an unpaired *t* test. Mann–Whitney U test was used to compare groups lacking normal distribution. For comparisons between paired groups, a paired *t* test or a Wilcoxon matched-pairs signed rank test was used for samples with or without normal distribution, respectively. Statistical analysis was carried out using GraphPad Prism Software v.8.0.1 (Prism, La Jolla, CA, USA) for Windows, and a *p*-value < 0.05 was considered significant.

## 3. Results

### 3.1. Confirmation of the Vagotomy Procedure

CCK-8 administration significantly reduced food intake in sham-vagotomized rats (0–1 h data: saline: 4.5 ± 0.4 vs. CCK-8: 2.1 ± 0.5 g; *p* < 0.001) rats, but not in vagotomized rats (0–1 h data: saline: 3.3 ± 0.4 g vs. CCK-8: 2.3 ± 0.4 g; *p* > 0.05). Sham-vagotomized rats’ stomach weights were also significantly lower than vagotomized rats’ stomach weight (sham: 100.0 ± 1.8% vs. vagotomized: 1034.0 ± 0.1%, *p* < 0.001). Vagotomized rats that failed to meet both criteria established for successful vagotomy (*n* = 2) were excluded from the statistical analysis, therefore the surgical procedure had an 83% success rate with 0% mortality.

### 3.2. Body Weight and Food Intake

**Figure 1:**
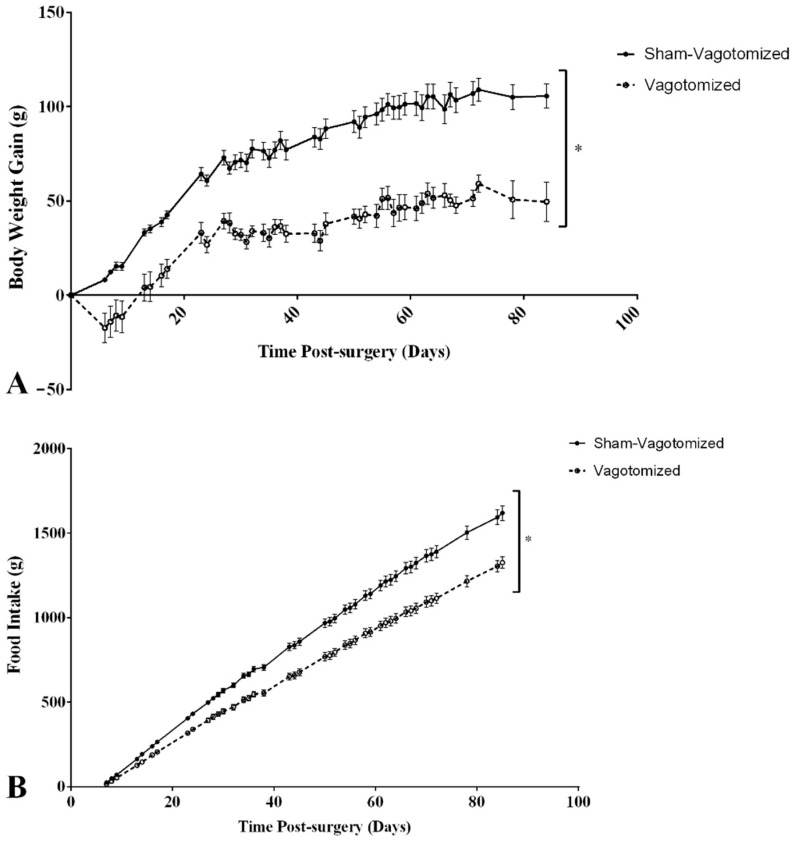
Body weight gain (**A**) and total food intake (from day 5 post-operation) (**B**) in confirmed vagotomized (*n* = 10) and sham-vagotomized rats (*n* = 12), expressed as mean ± SEM (* *p* < 0.05).

Body weight and cumulative body weight gain were significantly lower in the vagotomized group throughout the experimental period and at the endpoint (body weight: sham 386.8 ± 11.1 g vs. vagotomized 337.1 ± 9.7 g, *p* < 0.01; cumulative body weight gain: sham 105.5 ± 6.4 g vs. vagotomized 49.5 ± 10.4 g, *p* < 0.001). Daily food intake and cumulative food intake were significantly lower in vagotomized rats compared with sham vagotomized rats (cumulative food intake: sham 1618.9 ± 43.3 g vs. vagotomized 1326.0 ± 34.7 g, *p* < 0.001) (Figure 1A,B).

### 3.3. Feeding Response to Peripheral Acute GLP-1 Administration

**Figure 2:**
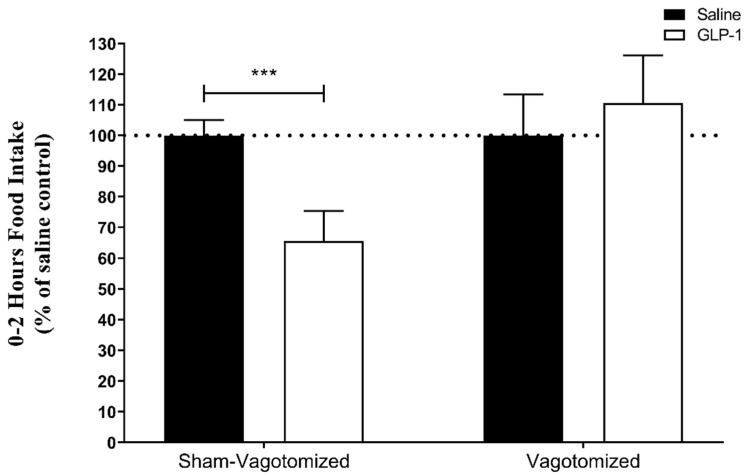
Effect on 0–2 h food intake of a single ip injection of saline (black bars) or 165 µg/kg GLP-1 (7–36) (white bars) administered during the early light phase to fasting sham-vagotomized (*n* = 12) or vagotomized rats (*n* = 10) expressed as mean ± SEM (*** *p* < 0.001).

GLP-1 (165 µg/Kg ip) significantly reduced food intake in sham-vagotomized rats (0–2 h after injection: saline: 7.0 ± 0.3 g vs. GLP-1: 4.6 ± 0.7 g; *p* = 0.001) rats, but not in vagotomized rats (0–2 h data: saline: 4.2 ± 0.6 g vs. GLP-1: 4.7 ± 0.7 g; *p* > 0.05) (Figure 2).

### 3.4. Adipose Tissue

**Figure 3:**
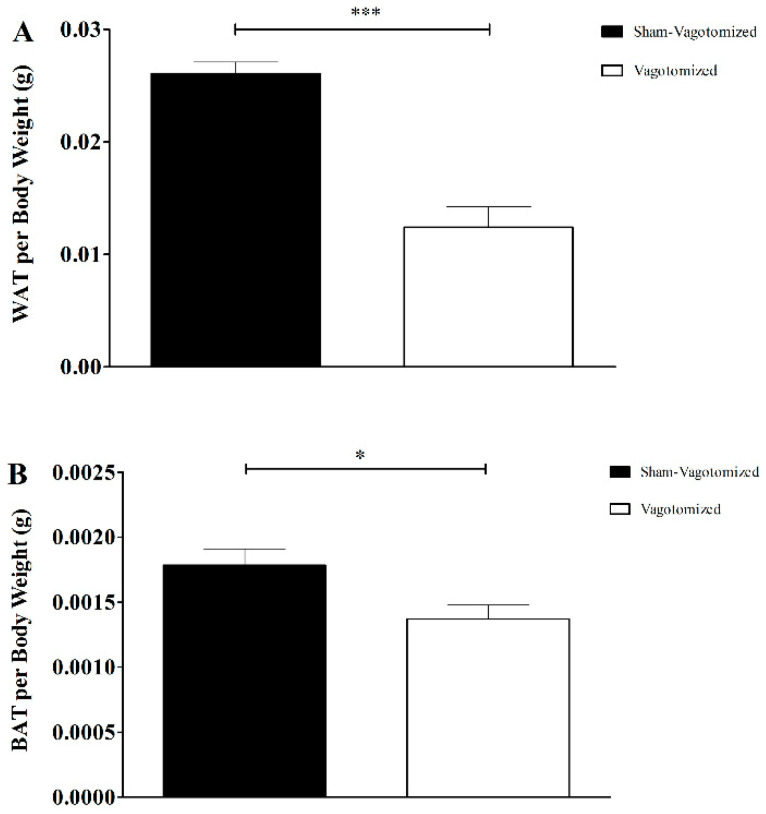
White adipose tissue (WAT) (**A**) and brown adipose tissue (BAT) (**B**) weight per gram of body weight of sham-vagotomized (*n* = 12) or vagotomized rats (*n* = 10). Results expressed as mean ± SEM (* *p* < 0.05; *** *p* < 0.001).

EPI WAT and BAT weight adjusted for total body weight of the rats was significantly lower in vagotomized rats (EPI WAT: sham 0.026 ± 0.001 g vs. vagotomized 0.012 ± 0.002 g; *p* < 0.001; BAT: sham 0.0018 ± 0.0001 g vs. vagotomized 0.0014 ± 0.0001 g, *p* < 0.05) (Figure 3A,B).

### 3.5. Resting Energy Expenditure

There was no significant difference in REE between the two groups of rats (sham 550.33 ± 41.65 J/24 h/kg vs. vagotomized 525.38 ± 35.25 J/24 h/kg; *p* > 0.05). No significant differences were found between any of the experimental groups regarding UCP-1 gene expression in BAT.

### 3.6. Hypothalamic Gene Expression

No significant differences were found between any of the experimental groups regarding NPY/AgRP and POMC/CART hypothalamic gene expression (Table S2).

### 3.7. Glucose and Hormone Plasma Levels

#### 3.7.1. Fasting Blood Glucose, Plasma Insulin Levels and HOMA-IR

Fasting blood glucose levels were significantly lower in vagotomized rats when compared to sham-vagotomized rats (sham 175.3 ± 78.2 mg/dL vs. vagotomized 91.6 ± 23.3 mg/dL; *p* < 0.05); however, when rats were injected with GLP-1, 90 min prior to sacrifice, no differences were found in glucose levels between the groups (sham 126.3 ± 49.1 mg/dL vs. vagotomized 122.8 ± 46.1 mg/dL; *p* > 0.05). Insulin levels were significantly lower in vagotomized rats in comparison to sham-vagotomized rats when injected with saline (sham 1.5 ± 0.3 ng/mL vs. vagotomized 0.5 ± 0.1 ng/mL; *p* < 0.01). After acute GLP-1 administration, there were no significant differences in insulin levels between the two groups (sham 1.4 ± 1.2 ng/mL vs. vagotomized 1.8 ± 1.4 ng/mL; *p* > 0.05). HOMA-IR was significantly lower in vagotomized rats (sham 16.4 ± 7.7 vs. vagotomized 2.8 ± 1.2; *p* < 0.05). HOMA-IR was not significantly modified by GLP-1 administration in either experimental group (sham: saline 16.4 ± 7.7 vs. GLP-1 10.7 ± 8.4; *p* < 0.05; vagotomized: saline 2.8 ± 1.2 vs. GLP-1 11.0 ± 6.5; *p* < 0.05) (Table 1).

#### 3.7.2. GI Hormones

Fasting total ghrelin levels were significantly higher in vagotomized rats when compared to the sham group (sham 1.0 ± 0.3 ng/mL vs. vagotomized 2.1 ± 0.7 ng/mL; *p* < 0.01), although no significant difference between the groups was observed after GLP-1 administration (sham 0.8 ± 0.3 ng/mL vs. vagotomized 1.4 ± 0.7 ng/mL; *p* > 0.05) (Table 1). Total and active GLP-1 levels were significantly higher in rats that received GLP-1, but no differences were found between rats of both groups when comparing saline- or GLP-1-injected animals. No differences were found in active/total GLP-1 ratio in the experimental groups (Table 1). No differences in PYY and OXM plasma levels were found between the experimental groups (sham saline-injected vs. sham GLP-1-injected or vagotomized saline-injected vs. vagotomized GLP-1-injected) (Table 1).

#### 3.7.3. Leptin

**Figure 4:**
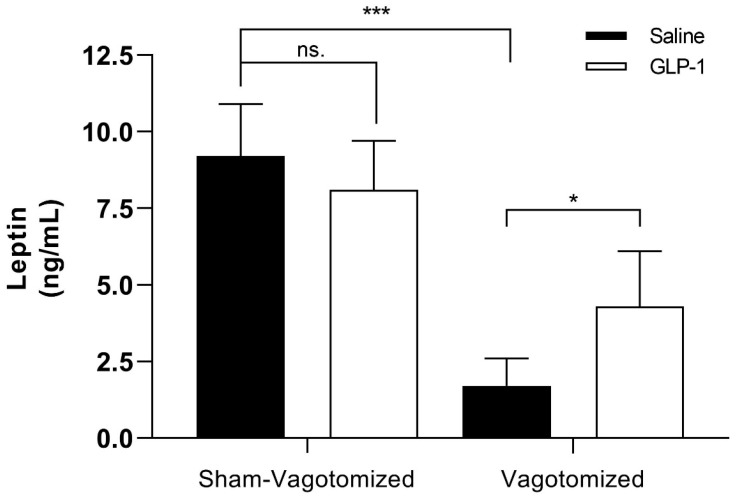
Leptin plasma levels (ng/mL) on sham-vagotomized or vagotomized rats 90 min after a single ip injection of saline or 165 µg/kg GLP-1 (7–36) administered during the early light (results are expressed as mean ± SEM (* *p* < 0.05 and *** *p* < 0.001). ns.-Non-significant.

Fasting leptin levels were significantly lower in vagotomized rats, both in the acute GLP-1- or saline-administrated, compared with their sham-operated counterparts (saline group: sham 9.2 ± 1.7 ng/mL vs. vagotomized 1.7 ± 0.9 ng/mL; *p* < 0.001; GLP-1 group: sham 8.1 ± 1.6 ng/mL vs. vagotomized 4.3 ± 1.8 ng/mL; *p* < 0.001). Conversely, vagotomized rats showed significantly higher leptin plasma levels after GLP-1 administration when compared with vagotomized rats administered with saline (vagotomized rats: saline 1.7 ± 0.9 ng/mL vs. GLP-1 4.3 ± 1.8 ng/mL; *p* < 0.01) (Figure 4).

### 3.8. In Vitro Stimulation of Human VAT Explants with GLP-1

VAT stimulation with 100 nmol/L of GLP-1 resulted in no significant differences in leptin secretion, as compared to blank controls, in any of the experimental groups. (Figure S1).

## 4. Discussion

In the present study, the role of the vagus nerve in whole-body energy homeostasis and in mediating the acute response to GLP-1 was evaluated. This study demonstrated that acute GLP-1 administration raises circulating leptin levels in truncally vagotomized rats but not in animals with an intact vagus nerve, thus suggesting that under physiological conditions, GLP-1-leptin dynamics rely on the integrity of gut–brain vagal pathway.

Rats that undergo truncal vagotomy are known to display lower food intake and present a distinctive body phenotype. However, the mechanisms by which the vagotomy procedure modifies whole-body energy homeostasis are complex and still not fully understood [25,26]. One of the mechanisms initially proposed for the phenomena was through energy expenditure modulation via BAT activation, a hypothesis that is not supported by our study data given that truncal vagotomy did not modify resting energy expenditure nor BAT UCP-1 expression. Non-shivering thermogenesis in BAT depends on UCP-1, a protein responsible for uncoupling proton leakage from ATP production to generate heat [27]. In humans, vagal stimulation was shown to increase energy expenditure via BAT activation [28]. In contrast, animal studies yielded mixed results, as vagal stimulation was shown to increase energy expenditure and UCP-1 expression in one study [29], while was reported to inhibit BAT thermogenesis activation in another study [30]. Therefore, the vagus nerve is more likely to serve as a conveyor to other signaling molecules that are able to modify food intake and glucose availability [31] than to regulate energy balance in a direct manner.

Vagotomized rats were also shown to present lower fasting glucose and insulin levels, which translates into a lower insulin resistance, as assessed by the homeostasis model. Given that vagotomized rats have lower food intake and body weight, glucose excursion and stimulus for insulin release are potentially decreased. Moreover, the impaired parasympathetic activity in vagotomized rats results in compromised adipogenesis and decreased adipose tissue mass, as previously documented [32]. Leptin is a hormone predominantly derived from WAT. Leptin enhances insulin sensitivity by acting peripherally, directly on tissues such as the skeletal muscle [33], but also through CNS action [34]. Indeed, insulin and leptin are well-recognized adiposity signals, as both are reported to vary in direct proportion to WAT reserves, while a lower adipose tissue mass is also reported to be associated with improved insulin and leptin sensitivity [35]. The higher fasting ghrelin levels observed in vagotomized rats are also consistent with the animals’ lower body weight, as fasting ghrelin is usually inversely correlated with the adiposity degree [36].

GLP-1 is a gastro-intestinal hormone that acts on the central nervous system (CNS) to decrease food intake and gastrointestinal motility. The GLP-1 anorectic effects were previously demonstrated to depend on the integrity of the vagus nerve [2]. Truncal vagotomy impaired the feeding inhibitory effects of acute GLP-1 administration, as in our study no significant reduction in food intake occurred in vagotomized rats, as observed in controls. A blunted GLP-1 anorectic effect was also witnessed in humans previously submitted to vagotomy for peptic ulcer treatment [37]. The mechanisms by which the vagus nerve is able to convey the GLP-1 signal to the CNS are far from being entirely known. GLP-1R activity in vagal afferent neurons were recognized to be crucial to mediate the effects of endogenous GLP-1 on food intake [38], but not necessarily for GLP-1 analogues that seem to act directly on the hypothalamic neurons [39]. In addition, GLP-1 is responsible for attenuating gastric emptying and gastro-intestinal motility, a mechanism that also depends on vagus nerve integrity [40,41]. After truncal vagotomy, gastric motility is maximally impaired as a result of severing the motor component of the vagus nerve that leads to secondary gastroparesis, thus further contributing to reduced food intake.

In human subjects, liraglutide, a GLP-1 analog, is well-documented to decrease leptin levels and reduce body weight [42], a phenomenon that is largely attributed to fat loss [43,44].The same was observed in vagotomized rats after chronic GLP-1 administration [21]. Moreover, treatment with liraglutide was also documented to be able to diminish the decrease in free plasma leptin during maintenance of weight loss, which possibly contributes to the weight maintenance effects [45].

PYY and OXM are anorectic peptide hormones secreted by intestinal L cells, which are also responsible for GLP-1 synthesis. Furthermore, GLP-1 was reported to suppress peptide hormone release by intestinal L cells and decrease PYY levels [46]. However, in our study, fasting PYY and OXM levels were not affected by vagotomy nor acute GLP-1 administration. These findings could be partially attributed to the fact that rats were only acutely exposed to GLP-1, a hormone known to have a very short half-life, and thus chronic stimulation is likely necessary to produce such down-regulation.

In the CNS, GLP-1 was previously reported to suppress NPY and AgRP gene expression, while increasing POMC and CART gene expression in 48 h fasting rats after central administration [47]. In our study, hypothalamic NPY, AgRP, POMC and CART gene expression were not modified by vagotomy nor GLP-1 administration, however, there are major methodological differences between the studies that do not render results comparable.

In the herein study, we demonstrated that vagotomized rats showed higher leptin levels after acute GLP-1 administration, suggesting that the effect of these two hormones on energy homeostasis are aligned in a manner that depends on the vagal pathway integrity. The role of the vagus nerve in mediating the leptin effects was also previously demonstrated. Deletion of leptin receptors from vagal afferent neurons in female mice results in an obese phenotype [48]. In turn, obesity leads to vagal dysfunction, which specifically hampers leptin response [49]. Furthermore, GLP-1R agonists were demonstrated to potentiate the anorectic effects of leptin by acting on the CNS [50], reinforcing the synergic effects of GLP-1 and leptin [51]. GLP-1R agonist weight loss effects are lost in leptin-resistant *db*/*db* mice, suggesting that GLP-1 anorexigenic effects are linked with leptin signaling [52]. GLP-1 is known to exert direct effects on white adipocytes that express GLP-1R [53]. GLP-1 down-regulates adipocytes fatty acid synthase [54], which is essential for fatty acid synthesis. Furthermore, GLP-1 analogs were demonstrated to stimulate lipid oxidation enzymes [55]. Free fatty acids are known to inhibit leptin secretion [56], thus we can hypothesize that GLP-1 could stimulate leptin release through fatty acid synthase inhibition and increased lipid oxidation, thus providing a possible explanation for our experimental findings. Moreover, the fact that this effect was only observed in vagotomized rats could be either attributed to a masking effect derived from adiposity or circulating adiposity signals such as insulin and leptin [57], or alternatively to the loss of a putative inhibitory vagal component that warrants further investigation. Furthermore, given that direct stimulation of VAT with GLP-1 was not shown to have any significant effect on leptin secretion, this suggests that the systemic effects on leptin levels are likely to be the end result of other indirect mechanisms.

This study has some limitations that should be taken into consideration while interpreting the data. From a methodologic standpoint, the fact that a neuronal tracer, a gold standard in assessing to what extent the vagal pathway was disrupted, was used, does not allows us to ensure that all procedures resulted in truncal and not subtotal vagotomies, nor exclude the possible partial nerve regeneration, which could have biased the results. Furthermore, given the fact that no further mechanistic studies were conducted on the possible mechanisms leading to enhanced leptin secretion, this remains a question that still needs to be addressed in order to be answered.

In summary, this study shows that after truncal vagotomy, rats depict a distinctive phenotype and response to acute GLP-1 administration. Overall, these study data confirm that the vagus nerve is able to modulate whole-body energy homeostasis by influencing food intake, body weight and body composition. The higher leptin levels observed in response to acute GLP-1 administration in truncal vagotomized rats further suggest the existence of a putative GLP-1-leptin axis that relies on the integrity of gut–brain vagal pathway.

## Figures and Tables

**Table 1:** Fasting glucose, insulin, HOMA-IR and GI hormone levels on successfully vagotomized and sham-vagotomized rats, 90 min after saline or GLP-1 administration.

	Sham-Vagotomized		Vagotomized	
	Saline (*n* = 6)	GLP-1 (*n* = 5)	Saline (*n* = 6)	GLP-1 (*n* = 5)
Glucose (mg/dL)	175.3 ± 78.2	126.3 ± 49.1	91.6 ± 23.3 *	122.8 ± 46.1
Insulin (ng/mL)	1.5 ± 0.3	1.4 ± 1.2	0.5 ± 0.1 **	1.4 ± 0.8
HOMA-IR	16.4 ± 7.7	10.7 ± 8.4	2.8 ± 1.2 *	11.0 ± 6.5
PYY (ng/mL)	0.3 ± 0.2	0.3 ± 0.1	0.2 ± 0.1	0.2 ± 0.1
OXM (ng/mL)	2.1 ± 1.6	2.2 ± 1.0	2.2 ± 1.7	2.0 ± 1.2
GLP-1 Active (pmol/L)	3.8 ± 0.4	62.1 ± 0.9 ***	3.8 ± 0.1	39.7 ± 0.1 ^†††^
GLP-1 Total (pmol/L)	24.1 ± 11.1	434.9 ± 172.7 **	21.8 ± 7.1	459.2 ± 352.6 ^†^
Active/Total GLP-1	0.18 ± 0.03	0.17 ± 0.04	0.16 ± 0.03	0.11 ± 0.03
Ghrelin (ng/mL)	1.0 ± 0.3	0.8 ± 0.3	2.1 ± 0.7 **	1.4 ± 0.7

Results expressed as mean ± SEM. *, ^†^ *p* < 0.05; ** *p* < 0.01; ***, ^†††^ *p* < 0.001; * vs. sham-vagotomized saline; ^†^ vs. vagotomized saline. HOMA–IR—homeostatic model assessment for insulin resistance; GLP-1—glugacon-like peptide-1; Oxm—oxyntomodulin; PYY—peptide YY.

## Data Availability

The data presented in this study are available upon request from the corresponding author due to ethical concerns.

## Supplementary Materials

The following supporting information can be downloaded at: https://www.mdpi.com/article/10.3390/biomedicines11051322/s1, Figure S1. Effect of GLP-1 100 ng/L stimulation on VAT leptin secretion across body mass index (BMI) and glycemic status. Results expressed as mean ± SEM; Table S1. Study subjects’ anthropometric, clinical and biochemical features.; Table S2. Relative hypothalamic gene expression of NPY/AgRP and POMC/CART on sham- and vagotomized rats 90 min after saline or GLP-1 administration.
