# Supplementary material for: GLP-1 Increases Circulating Leptin Levels in Truncal Vagotomized Rats
Source: Biomedicines. 2023 Apr 28;11(5):1322. doi: 10.3390/biomedicines11051322 (PMC10216183; doi:10.3390/biomedicines11051322)
Supplement: Supplementary file 1 [file biomedicines-11-01322-s001.zip › Table S1.pdf]

Table S1. Study subjects' anthropometric, clinical and biochemical features.

	Ob+NGT (n = 5)	Ob+Pre-T2D (n = 5)	Ob+T2D (n = 5)	Non-Ob (n = 4)
Age (years)	44 ± 7	50 ± 3	56 ± 2	48 ± 7
Sex (F%)	80%	80%	80%	50%
BMI (kg/m^2)	41.4 ± 2.6 ***	44.0 ± 2.8 ***	41.5 ± 2.5 ***	26.1 ± 1.0
Fasting glucose (mg/dL)	93.8 ± 0.8	109.2 ± 8.0	161.4 ± 26.8 *,†	88.8 ± 3.2
HbA1c (%)	5.4 ± 0.2	6.2 ± 0.2	7.6 ± 1.3 †	n.a.
Metformin (%)	0%	0%	100%	0%
SBP (mmHg)	137 ± 7	150 ± 6	146 ± 4	133 ± 7
DBP (mmHg)	79 ± 4	85 ± 3	73 ± 4	80 ± 4
Total cholesterol (mg/dL)	161 ± 7	220 ± 15	206 ± 24	177 ± 25
HDL (mg/dL)	52 ± 5	47 ± 7	48 ± 5	35 ± 11
LDL (mg/dL)	96.4 ± 10.3	142.4 ± 7.2	135.4 ± 23.2	100.9 ± 6.7
Triglycerides (mg/dL)	102 ± 8	111 ± 22	203 ± 49	206 ± 147

Subjects were grouped according to body mass index (BMI) and glycemic status (with obesity and euglycemia—Ob+NGT; with obesity and pre-diabetes—Ob+Pre-T2D; with obesity and T2D—Ob+T2D; without obesity—Non-Ob). HbA1c—hemoglobin A1c; SBP—systolic blood pressure; DBP—diastolic blood pressure; HDL—high-density lipoprotein; LDL—low-density lipoprotein. Data are presented as mean ± SEM. * vs. Non-Ob (*, $p < 0.05$; ***, $p < 0.001$); † vs. Ob+NGT (†, $p < 0.05$).
