# Supplementary material for: GLP-1 Increases Circulating Leptin Levels in Truncal Vagotomized Rats
Source: Biomedicines. 2023 Apr 28;11(5):1322. doi: 10.3390/biomedicines11051322 (PMC10216183; doi:10.3390/biomedicines11051322)
Supplement: Supplementary file 1 [file biomedicines-11-01322-s001.zip › Table S2.pdf]

Table S2. Comparison of hypothalamic gene expression of NPY/AgRP and POMC/CART on sham- and vagotomised rats 90 minutes after Saline or GLP-1 administration.

	**Sham-Vagotomised**		**Vagotomised**	
	**Saline (n=6)**	**GLP-1 (n=6)**	**Saline (n=5)**	**GLP-1 (n=5)**
**NPY**	1.00±0.11	1.02±0.14	1.51±0.25	1.51±0.25
**AgRP**	1.00±0.33	1.15±0.39	0.75±0.67	0.61±0.39
**POMC**	1.00±0.22	1.13±0.40	0.93±0.45	1.04±0.38
**CART**	1.00±0.38	1.02±0.35	1.40±0.44	0.96±0.30
**UCP-1**	1.00±0.24	0.86±0.19	0.52±0.15	0.87±0.29
