# Supplementary figures and images for: GLP-1 Increases Circulating Leptin Levels in Truncal Vagotomized Rats
Source: Biomedicines. 2023 Apr 28;11(5):1322. doi: 10.3390/biomedicines11051322 (PMC10216183; doi:10.3390/biomedicines11051322)

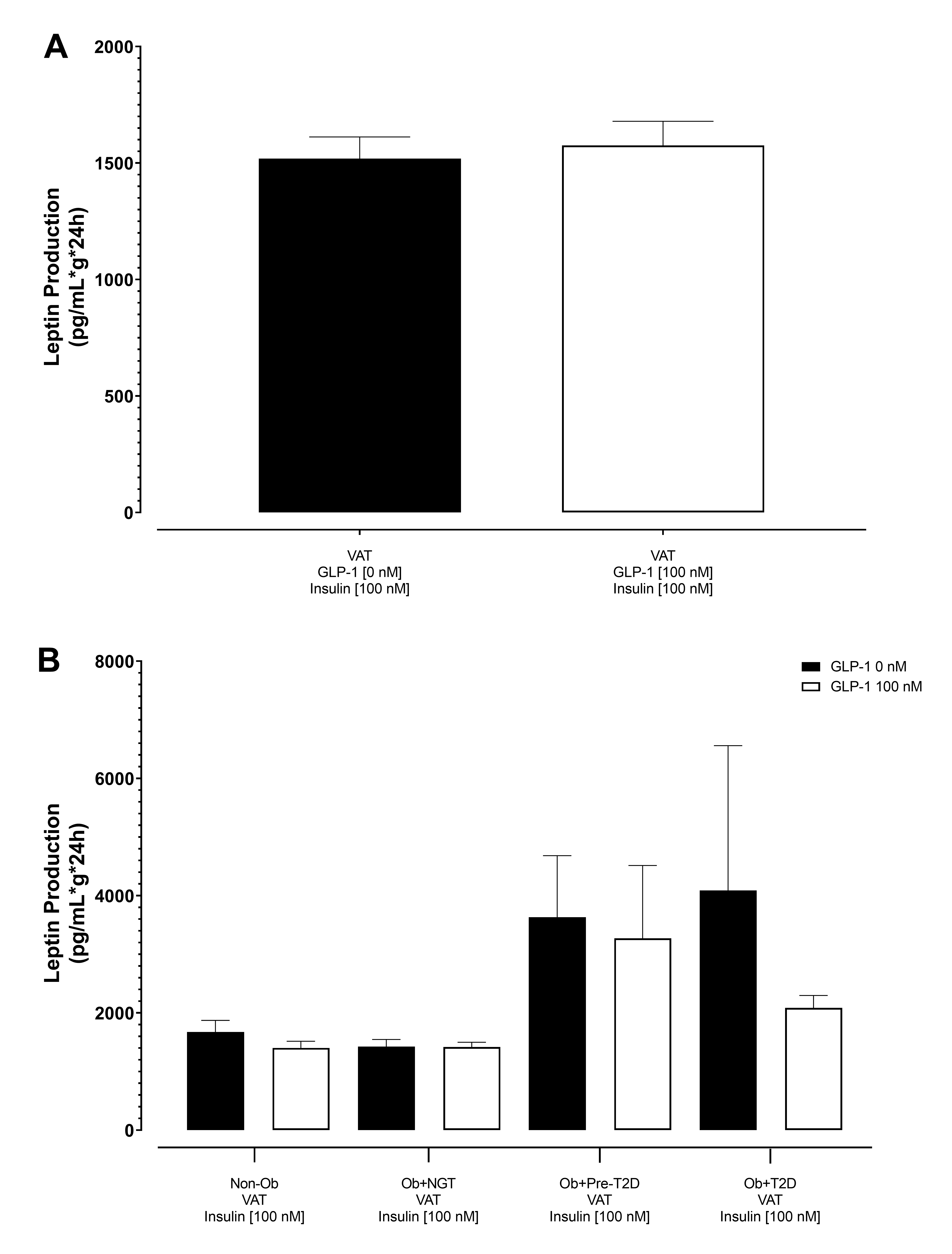

Supplement: Supplementary file 1 [file biomedicines-11-01322-s001.zip › Figure S1.tif]
